# Arthroscopic Three Hybrid Anchor Construct Fixation in Skeletally Immature Patients With Tibial Eminence Fractures

**DOI:** 10.1002/atn2.70019

**Published:** 2026-04-28

**Authors:** Chaofan Liao, Peidong Liu, Qiuzhen Liang, Bohao Zhang, Penghui Cui, Jiang Zheng, Liang Zhang

**Affiliations:** ^1^ Sports Medicine Center Honghui Hospital Xi'an Jiaotong University Xi'an China

## Abstract

This article describes a surgical technique for skeletally immature patients with tibial eminence fractures of the anterior cruciate ligament (ACL) insertion. Owing to the distinct anatomical characteristics in skeletally immature patients compared with adults, we arthroscopically implanted a three hybrid anchor construct into the posterior, anteromedial, and anterolateral aspects of the fracture bed, respectively, so that the force of the downward fracture fragment was distributed in a triangular network with more uniform force. The main advantages of this method are that there is no need to drill any transphyseal bone tunnel, the anchors are implanted in the proximal tibial physis to avoid damage to the physis and ACL, and all operations are performed under arthroscopy.

## Graphical abstract

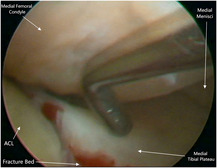

**VIDEO 1:** Surgical technique of the three hybrid anchors. Video content can be viewed at https://doi.org/10.1002/atn2.70019.

Avulsion fractures of the anterior cruciate ligament (ACL) tibial insertion are most common in children and adolescents aged 8 to 14 years, with an annual incidence of 3/100,000, accounting for approximately 2% to 5% of knee injuries in children.^1^ Because the mechanical strength of the growth plate–metaphyseal bone interface is lower than that of ligaments, epiphyseal fractures or epiphyseal injuries are more frequent in young children.^2^ Currently, for Meyers and McKeever type 2, 3, and 4 of tibial eminence fractures (TEF), it has been generally believed that arthroscopy or open reduction internal fixation should be performed to fix the fracture fragment and restore the stability of the knee joint.^3^, ^4^, ^5^ However, there are still some disadvantages according to the traditional adult surgical method or the pediatric way.^6^ Therefore, we describe a technique using three anchors to disperse the force uniformly on the fracture fragment, with no bone tunnel drilling and additional auxiliary portals (Video 1).

## SURGICAL TECHNIQUE

### Position and Portals

**FIGURE 1:**
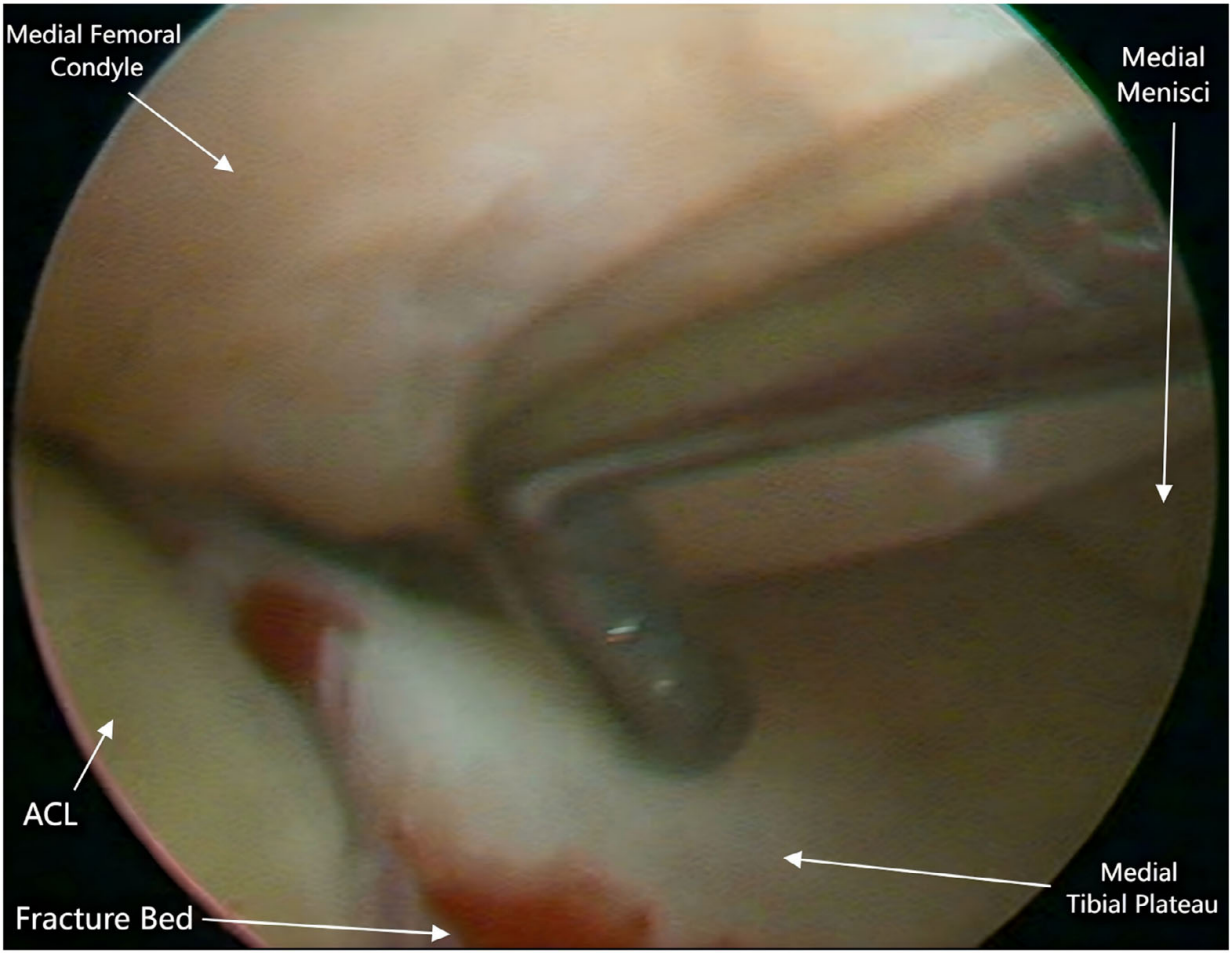
View from the anteromedial portal of a right knee with a probe to explore the structures (including hemarthrosis, menisci, articular cartilage, and ligaments) in the joint cavity.

The patient is placed in the supine position with a tourniquet on the lower limb and sterilized with a drape. Conventional anteromedial (AM) and anterolateral (AL) arthroscopic portals are established with the knee in 90° of flexion inside the waterproof bag. The whole joint is examined for combined intra‐articular injuries, including hemarthrosis, menisci, articular cartilage, and ligaments. Proliferated soft tissue and hematoma are removed to make the surgical field clearer. Then, the anterior horn of the menisci and small fracture fragments are probed carefully, which would affect the reduction of fractures (Figure 1). Subsequently, the fracture bed of the tibial crater is debrided as much as possible when necessary.

### Reduction and Fixation

**FIGURE 2:**
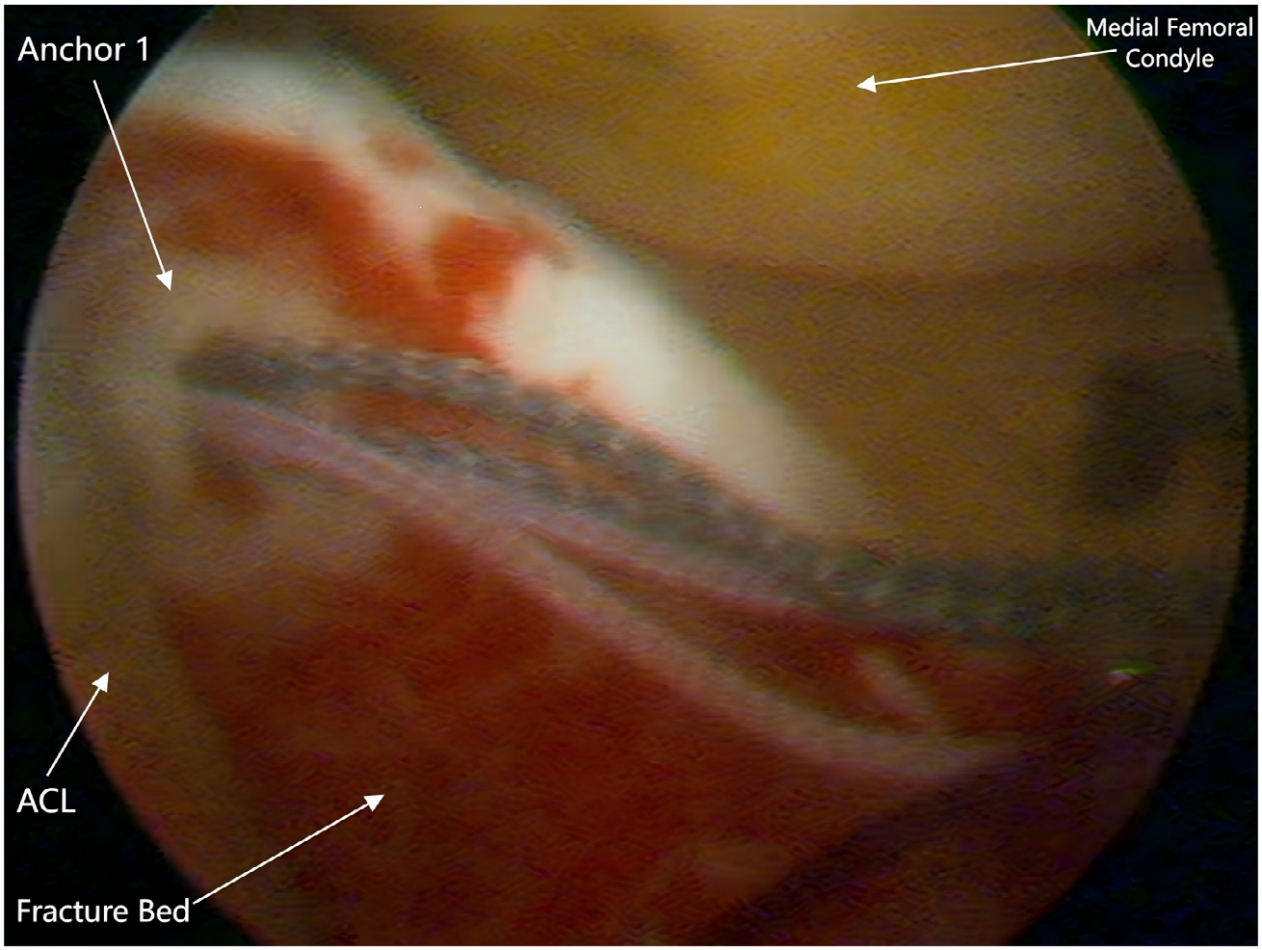
View from the anteromedial portal of a right knee. The first anchor (anchor 1) is screwed from anteromedial portal to the inner posterior edge of the fracture bed, with a direction 45° distally and axial alignment to the lower extremity.

**FIGURE 3:**
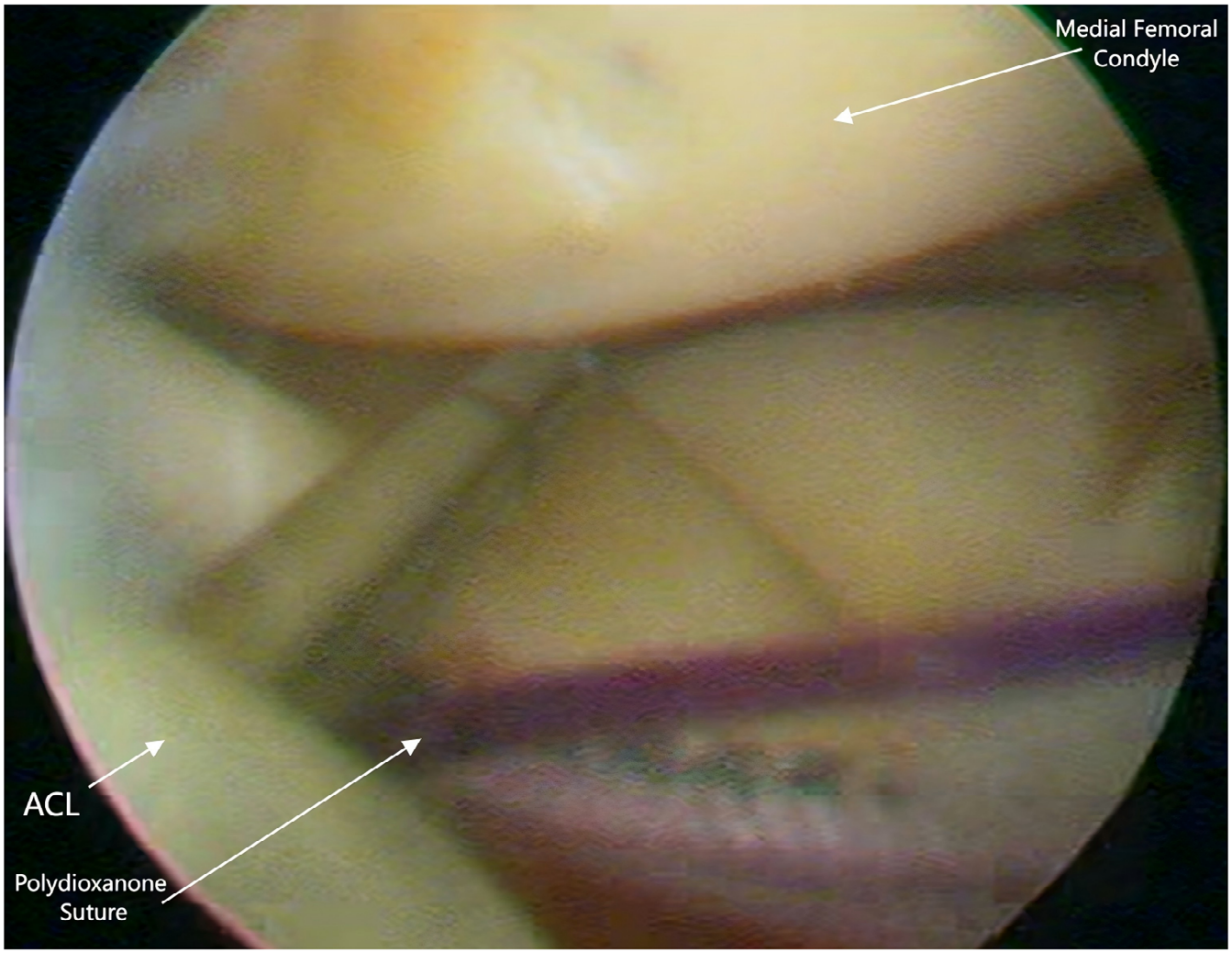
View from the anteromedial portal of a right knee. A polydioxanone suture is used to partially cross the distal end of the ACL with a hook.

**FIGURE 4:**
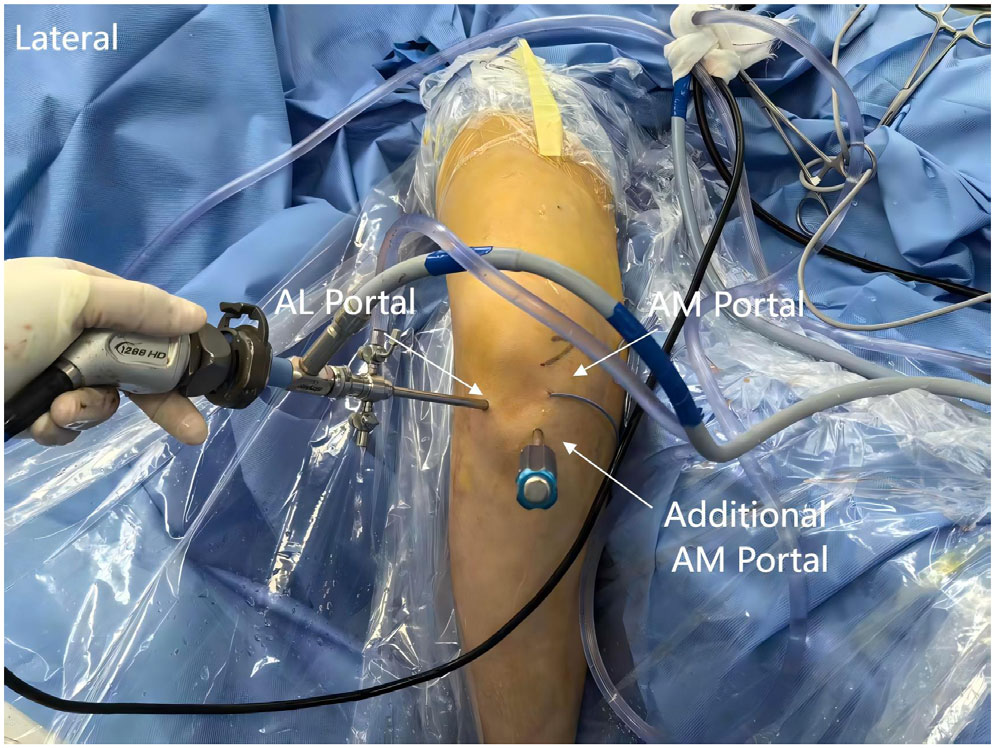
Clinical photograph of a right knee. An additional AM portal is established in the more distal and lateral position of the standard AM portal. (AM, anteromedial; AL, anterolateral.)

**FIGURE 5:**
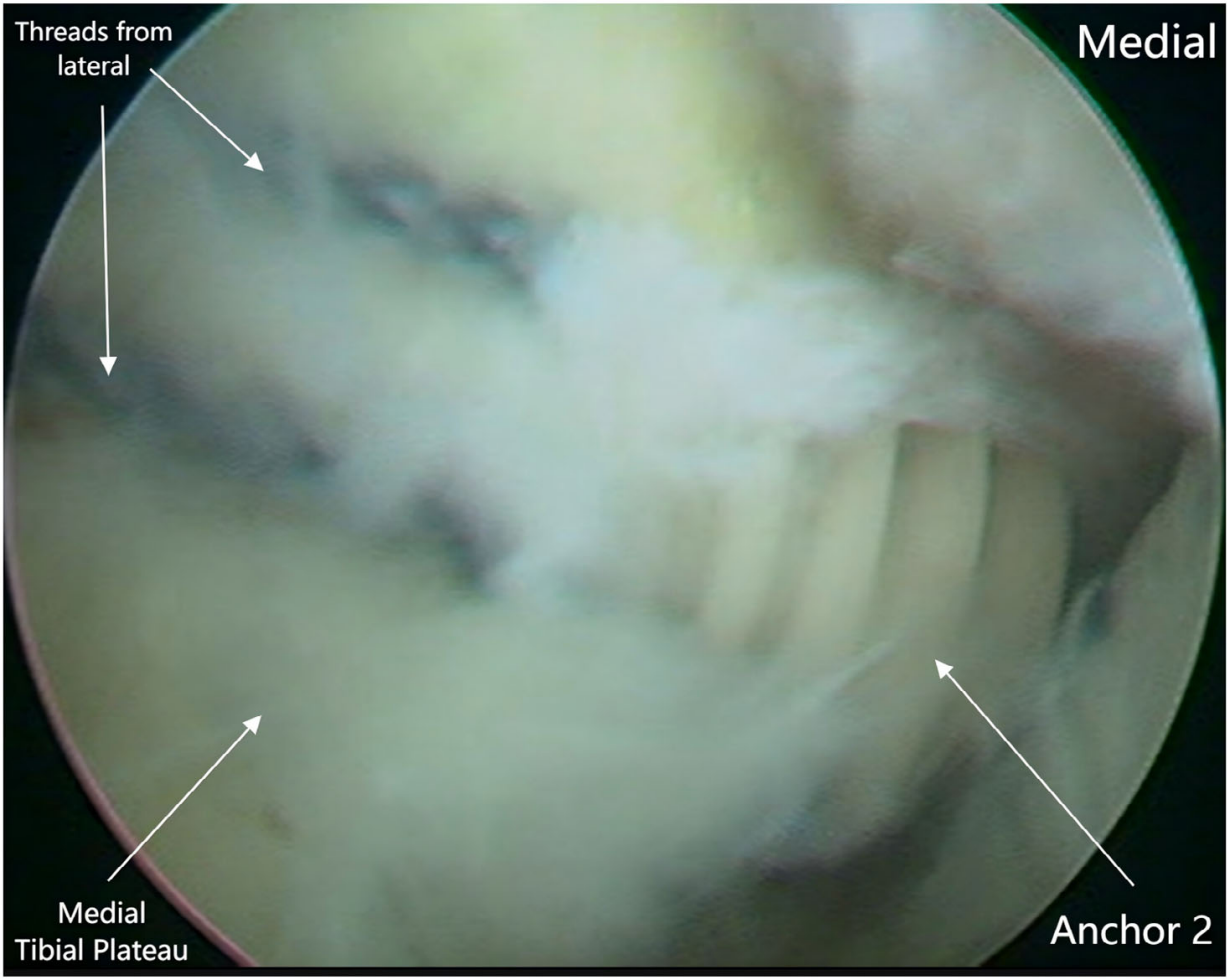
View from the additional anteromedial portal of a right knee. The second and third anchor (anchor 2 and anchor 3) are screwed to the tibial plateaurespectively, with a direction 45° distally and axial alignment to the lower extremity.

**FIGURE 6:**
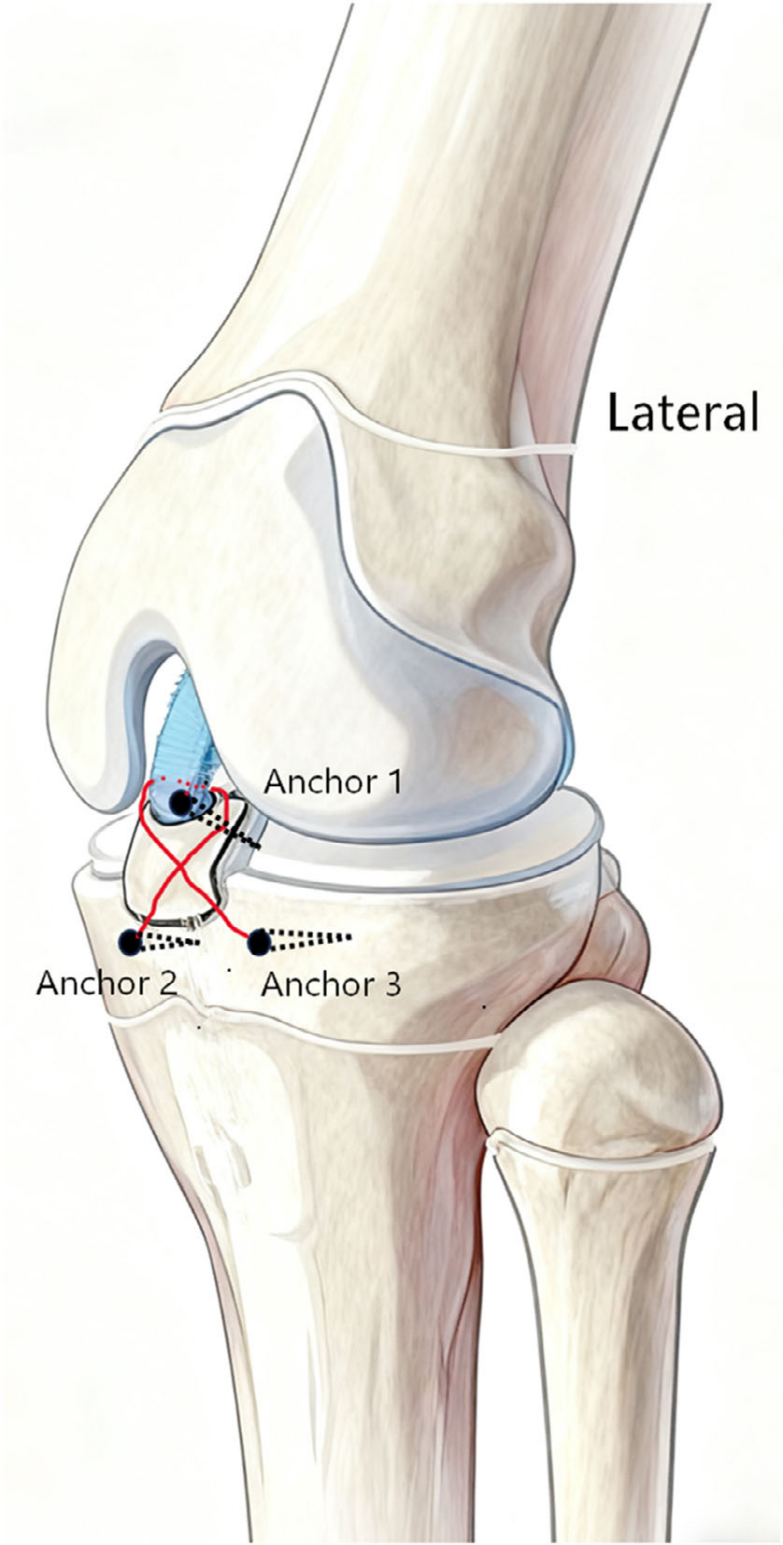
The position and direction of three hybrid anchors.

The intercondylar fossa is observed via the AL portal, and a probe is used from the AM portal to attempt the reduction of the fracture fragment, plan and determine the position of anchors, then mark it with a shaver. The first anchor (BIORAPTOR CURVED PK Suture Anchor, 2.3 mm, Smith & Nephew, Andover, Massachusetts, USA) is screwed into the premarked point (Figure 2), which is immediately adjacent to the inner posterior edge of the fracture bed, with a direction 45° distally and axial alignment to the lower extremity (anchor 1). Intraoperative X‐ray fluoroscopy is performed to ensure that the anchor position is on the proximal epiphyseal. A polydioxanone suture is used through the distal part of the ACL with a hook (Figure 3). The threads of anchor 1 are pulled around the front of the ACL from the outside, and then the threads are pulled around the back of the ACL from the inside in the same way. Next, an additional AM portal is established in the more distal and lateral position of the standard AM portal (Figure 4), taking care to protect the anterior horn of the meniscus and the intermeniscal ligament. A shaver is inserted from the additional AM portal to clean the soft tissue in front of the tibial plateau in order to obtain a good view. Combined with intraoperative fluoroscopy, the second anchor (FOOTPRINT Ultra PK Suture Anchor, 4.5 mm, Smith & Nephew, Andover, Massachusetts, USA) is implanted on the AM side of the tibial plateau (Figure 5), with a horizontal or slightly distal direction (anchor 2). Similarly, the third anchor (FOOTPRINT Ultra PK Suture Anchor, 4.5 mm, Smith & Nephew, Andover, Massachusetts, USA) is placed on the AL side of the tibial plateau, in a similar direction (anchor 3). The thread tail from the previously implanted anchor 1 is passed through the medial and lateral two suture anchor (anchor 2 and 3), respectively, and held in place; then the residual thread tail is cut (Figure 6). Finally, the intercondylar fossa is observed as a whole again, and a probe is used to check the tension of the ACL.

### Postoperative Rehabilitation

After surgery, the knee is placed in a hinged knee brace and locked for an extension of 2 weeks. Ankle pumps, straight leg raises, and other active and passive knee movements should be allowed as early as possible. After 2 weeks, the angle of the brace is adjusted according to the situation to increase the range of motion of the knee joint, the closed chain activity is added appropriately, and the patient is allowed to partially bear weight with a cane, then full weight bearing at 4 weeks. Six weeks later, the range of motion, stability of the knee joint, and the strength of the quadriceps femoris will be evaluated, which determines whether the patient can return to sports.

## DISCUSSION

TEF in skeletally immature patients presented unique surgical challenges due to the risk of epiphysis injury. While multiple fixation methods exist, significant limitations persist: open reduction internal fixation with metal screw or Kirschner wires necessitates extensive dissection and increases soft tissue trauma. Moreover, screw fixation risks fragment comminution in small fracture fragments. However, surgeons recently reported that the insertion and removal of metal screws or Kirschner wires could be performed arthroscopically instead of traditional open reduction internal fixation.^7^, ^8^ Crucially, metal screws or Kirschner wires always need secondary removal surgery.^3^ High‐strength suture often requires transtibial bone tunnels, which pose irreversible physeal damage risks, and studies reported growth disturbances in pediatric cases.^9^ Biomechanically, suture elongation with growth and development of young patients may lead to fixation failure. Anchor fixation includes single‐row, hybrid suture anchors and knotless anchors, which are the most common surgical method to TEF in children. However, it creates an uneven force distribution theoretically.^10^, ^11^

### Advantages of the Three Hybrid Anchor Construct

Minimally invasive arthroscopic execution utilizes only three 5‐mm portals (standard AL, standard AM, and additional AM), and reduces intra‐articular complication. Epiphysis preservation, because the preoperative computed tomography–guided trajectory planning enables anchor insertion at 45° distal angles and maintains >3 mm clearance from the physics. In addition, this technology does not require drilling bone tunnels, which significantly reduces the risk of injury. Biomechanical superiority by using triangular force distribution creates a stable tension band, which is critical for accommodating pediatric activity demands (Tables 1 and 2).

**TABLE 1:** Pearls and Pitfalls

Pearls	Pitfalls
Use proper portal for optimal view and could establish the additional auxiliary portal when necessary	Avoid excessive damage to the ACL during the suturing process
Check epiphyseal depth preoperatively with MRI or CT to avoid damage	Risk of suture failure if not properly tensioned
Place suture anchors at a 45° angle from the tibial plateau to spare the physis	Technical difficulty in suture management and anchor placement, requiring experience
Perform all‐inside repairs for additional stability in hybrid techniques	Inadequate reduction may lead to nonunion or instability

ACL, anterior cruciate ligament; CT, computed tomography; MRI, magnetic resonance imaging.

**TABLE 2:** Advantages and Disadvantages

Advantages	Disadvantages
Physical‐sparing, avoiding growth plate damage	Requires experience for TEF in arthroscopic surgery
Minimal invasion with small incisions and no tibial tunnels	Risk of suture failure or implant‐related issues
Complete anatomic reduction in a triangular network with uniform force	Not validated by biomechanical studies in technique
No intra‐articular hardware, eliminating need for second surgery	Limited patient cohort and need for further research
Economical and simple techniques with bone‐sparing approaches	Potential for iatrogenic osteochondral injury if anchor placement is inaccurate

TEF, tibial eminence fractures.

There are some limitations of this technique. This technique may damage the anterior horn of the meniscus, which is worthy of attention, because the anchor 2 and anchor 3 are located below. Special attention should be paid to the protection of the anterior horn of the meniscus and the intermeniscal ligament when getting the view of this part. Next, sometimes the ACL is damaged, because it needs to partially cross the distal end of the ACL when fixing the split fracture fragment. However, the choice can also be made according to the situation. If the fracture fragment is large and not split, it can be considered to simply use the threads from anchor 1 to bypass the ACL for binding and directly fix using anchor 2 and anchor 3. Finally, research is still necessary, including biomechanical analysis and multicenter, long‐term clinical follow‐up observations.

## DISCLOSURES

The authors (C.L., P.L., Q.L., B.Z., P.C., J.Z., L.Z.) declare that they have no known competing financial interests or personal relationships that could have appeared to influence the work reported in this paper.
